# Sexual delay discounting as a moderator of the association between substance use and condomless anal sex among young black and Latino men who have sex with men

**DOI:** 10.1016/j.abrep.2026.100731

**Published:** 2026-08-01

**Authors:** Rui Luo, Xiaoming Li, Xueying Yang

**Affiliations:** a Department of Health Promotion, Education, and Behavior, Arnold School of Public Health, University of South Carolina, Columbia, SC, USA; b South Carolina SmartState Center for Healthcare Quality, Columbia, SC, USA; c University of South Carolina Big Data Health Science Center, Columbia, SC, USA

**Keywords:** Men who have sex with men, Condomless anal sex, Sexual delay discounting, HIV, Substance use

## Abstract

**Background:**

This study examined sexual delay discounting (SDD) as a potential factor associated with the relationship between substance use and condomless anal sex (CAS) among Black and Latino men who have sex with men (MSM).

**Methods:**

Baseline data were drawn from a multi-site HIV testing promotion trial (NIDA-CTN-0083) involving Black and Latino MSM across eight U.S. states and the District of Columbia. Past 90-day CAS and past 12-month substance use were assessed via self-report. SDD was measured using the Sexual Delay Discounting Task and summarized as area under the curve (AUC) values. K-means clustering identified four SDD profiles: high self-control, globally impulsive, immediate-impulsive, and delayed-impulsive. Ordinal logistic regression models examined associations among substance use, SDD profiles, and CAS.

**Results:**

A total of 271 participants were included. Compared with the high self-control group, the odds of CAS were significantly higher among the delayed-impulsive (OR = 2.978, *p* = 0.001), immediate-impulsive (OR = 8.970, *p* < 0.001), and globally high-discounting groups (OR = 9.427, *p* < 0.001). Alcohol and illicit drug use were modeled separately. Although substance use showed no overall main effect, significant interactions indicated that illicit drug use was more strongly associated with CAS among immediate-impulsive (OR = 1.485, *p* = 0.039) and globally high-discounting participants (OR = 1.686, *p* = 0.004).

**Conclusions:**

SDD profiles were associated with CAS and may shape how illicit drug use relates to sexual risk behavior among Black and Latino MSM. These findings highlight the importance of considering temporal impulsivity in HIV prevention efforts.

## Highlights

• Four distinct sexual delay discounting profiles identified using clustering.
• Illicit drug use increases sexual risk only in delayed and globally impulsive groups.
• Temporal impulsivity identified as key mechanism for sexual risk.

## 1 Introduction

In the United States, young men who have sex with men (MSM) remain one of the populations most disproportionately affected by human immunodeficiency virus (HIV) infection. According to surveillance data from the Centers for Disease Control and Prevention (Control, C. f. D. and Prevention, 2025), MSM accounted for 66% of newly diagnosed HIV cases nationwide, representing approximately 25,740 individuals. Among these, Black/African American and Hispanic/Latino MSM together comprised more than one-third of new diagnoses, with Black/African American MSM alone accounting for 38% (14,754 cases), indicating marked racial and ethnic disparities (Control, C. f. D. and Prevention, 2025). In addition, individuals aged 25–34 years represented the largest proportion of new HIV diagnoses (31.3%), suggesting that early adulthood remains a critical high-risk period for HIV infection (Control, C. f. D. and Prevention, 2025). Condomless anal sex (CAS) continues to be recognized as the primary route of HIV transmission among MSM (Baggaley et al., 2010). Due to the fragility of rectal mucosal tissue, immunological susceptibility, and specific behavioral characteristics, the risk of HIV transmission through anal intercourse is substantially higher than that associated with heterosexual contact (Kelley et al., 2017).

A substantial body of research has demonstrated that substance use, including alcohol consumption, illicit drug use, and misuse of prescription medications, is closely associated with sexual risk behavior and significantly increases the likelihood of HIV infection(Berry & Johnson, 2018; Degenhardt et al., 2023; Hibbert et al., 2021). For instance, among MSM, individuals who engage in “chemsex” are more likely to have CAS and multiple sexual partners (Amundsen et al., 2024). However, it is important to note that not all people who use substances exhibit sexual risk behavior. Systematic reviews have revealed considerable heterogeneity in the association between substance use and sexual risk behaviors, suggesting that personal traits may play a critical moderating role (Maxwell et al., 2019). Consequently, researchers have begun to focus on the psychological mechanisms underlying sexual decision-making processes to better understand why substance use influences sexual risk behaviors differently across individuals (Maisto et al., 2012; Scott-Sheldon et al., 2016).

The theory of delay discounting offers an important framework for understanding such individual differences (Matta et al., 2012). This theory posits that when faced with choices between immediate and delayed rewards, individuals tend to devalue delayed rewards as the waiting time increases (Odum, 2011). In other words, those who find it more difficult to wait for future outcomes exhibit a greater discounting of delayed rewards, reflecting a stronger present bias (Bickel et al., 2014). In the context of sexual behavior, this concept has been extended to sexual delay discounting (SDD), which assesses the tendency of individuals to choose between immediate unprotected sex and waiting to obtain a condom for protected sex (Johnson and Bruner, 2012a, Johnson and Bruner, 2012b). By calculating the probability of choosing to wait under varying delay conditions, researchers can derive a discounting rate that reflects impulsiveness in sexual decision-making. A higher level of SDD reflects a stronger preference for immediate gratification and a greater propensity to engage in unprotected sex, whereas a lower level indicates greater self-control and a stronger inclination toward delayed gratification (Jarmolowicz et al., 2017).

An increasing number of studies have demonstrated a close association between SDD and sexual risk behavior. Individuals with higher SDD scores are more likely to report more frequent CAS, and a greater number of sexual partners (Collado et al., 2017; Hahn et al., 2019a, Hahn et al., 2019b; Johnson and Bruner, 2012a, Johnson and Bruner, 2012b). Furthermore, substance use has been found to further influence the delay discounting process: alcohol, illicit drugs, and other psychoactive substances can impair self-control and amplify the preference for immediate gratification, thereby elevating SDD levels and increasing the likelihood of CAS (Johnson et al., 2021; Johnson and Bruner, 2012a, Johnson and Bruner, 2012b). Thus, SDD not only reflects the individual's trade-off between immediate gratification and risk regulation in sexual contexts but may also serve as a key psychological mechanism linking substance use and sexual risk behaviors.

Although previous studies have confirmed a significant association between SDD and sexual risk behaviors, most research has primarily focused on its main effect, that is, the role of SDD as a direct predictor of unprotected sexual behavior (Dariotis and Johnson, 2015a, Dariotis and Johnson, 2015b; Gebru et al., 2022; Sweeney et al., 2020). However, sexual risk behaviors are typically shaped by multiple interacting factors, and analyses limited to main effects may fail to capture individual differences in responses to external stimuli such as substance use. Therefore, examining the moderating role of SDD in the relationship between substance use and sexual risk behavior is of critical importance for elucidating the psychological mechanisms underlying impulsive sexual decision-making. Moreover, existing studies have almost exclusively treated SDD as a continuous variable (; Hahn et al., 2019a, Hahn et al., 2019b), emphasizing linear individual differences while overlooking the possibility that individuals may exhibit qualitatively distinct sexual decision-making profiles. In reality, decision-making tendencies may differ across short- and long-delay contexts(Lukinova & Erlich, 2021; Zauberman et al., 2009). Identifying latent patterns of SDD can not only reveal within-group heterogeneity but also offer a more nuanced understanding of how impulsivity operates in sexual risk decision-making.

Building upon the existing research background and theoretical framework, this study focused on young racial and ethnic minority MSM to systematically examine the role of SDD in the association between substance use and sexual risk behavior. Specifically, the objectives were: (1) to examine the direct associations among SDD, substance use, and CAS; and (2) to explore the heterogeneity of SDD by identifying distinct decision-making groups and comparing their moderating effects.

## 2 Methods

### 2.1 Participants

Data for this study were drawn from the baseline survey of the NIDA-CTN-0083 project (Stafylis et al., 2022). Participants were young Black/African American and Latino MSM. Inclusion criteria were: (1) aged 18–30 years; (2) assigned male at birth; (3) history of sexual activity with men; (4) self-identification as Latino or Black/African American; and (5) reporting either CAS or more than one male sexual partner in the past 90 days. These criteria were designed to identify participants with elevated potential HIV exposure risk within sexual networks and were inherited from the parent study protocol. Exclusion criteria included: (1) self-reported HIV-positive status; (2) having undergone HIV testing within the past 90 days; (3) current use of pre-exposure prophylaxis (PrEP); and (4) participation in another HIV prevention-related study within the previous six months.

### 2.2 Procedure

This study is a secondary analysis of baseline data from a longitudinal observational cohort designed to promote free HIV self-testing through multiple online platforms(Stafylis et al., 2022). Recruitment advertisements were placed across three types of platforms, social media, dating applications, and search engines. Ads targeted U.S. regions with higher HIV prevalence, including the District of Columbia and eight states. Individuals who clicked the advertisement were directed to an informational webpage detailing the study objectives and procedures and were invited to complete an eligibility screening. Eligible participants who provided electronic informed consent were then asked to complete the baseline questionnaire assessment. This study was approved by the Institutional Review Board at the University of California, Los Angeles (IRB #18–001580).

### 2.3 Measurements

#### 2.3.1 CAS

CAS was assessed using the item: “During the past 90 days, how often did you use condoms during anal sex?” Responses were provided on a five-point Likert scale (1 = Always, 2 = Most of the time, 3 = About half the time, 4 = Sometimes, 5 = Never). Lower frequency of condom use indicated a higher risk of engaging in CAS.

#### 2.3.2 Substance use

Substance use was measured using the Tobacco, Alcohol, Prescription medication, and other Substance use (TAPS) tool (Health, 2026). This instrument assesses the frequency of alcohol, illicit drug, and prescription medication use during the past 12 months. Participants reported their use of substances such as alcohol (e.g., “In the past 12 months, how often did you have five or more alcoholic drinks in a single day?”), illicit drugs (e.g., marijuana, cocaine/crack, heroin), and prescription medications (e.g., opioid pain relievers, anti-anxiety/sleep aids, ADHD medications). Illicit drug use and prescription medication misuse were assessed as unified categories. Items were rated on a seven-point scale (1 = Never, 2 = Less than monthly, 3 = Monthly, 4 = Weekly, 5 = Daily or almost daily), with higher scores indicating more frequent substance use. The TAPS is a widely used screening instrument with demonstrated validity for assessing alcohol and substance use across adult populations.(Gryczynski et al., 2017).

### 2.4 SDD

SDD tendencies were assessed using the Sexual Delay Discounting Task (SDDT) (Johnson and Bruner, 2012a, Johnson and Bruner, 2012b; Johnson & Bruner, 2013), which evaluates impulsive decision-making in sexual contexts. Participants were shown 20 photographs of men with varying physical appearances and instructed to select the individual with whom they would most like to have sex, based solely on appearance. All subsequent delay discounting judgments were made with respect to this selected partner. Participants were instructed to imagine that the partner's personality matched their preferences and that the situation was appropriate for sexual activity. They were further instructed to assume that the partner's STI/HIV status was unknown. To improve cultural relevance, the photographic stimuli used in the SDDT had been adapted by the original study investigators to better reflect the preferences and characteristics of the target population.

The task included eight scenarios: conditions where condoms were immediately available and those where condoms were temporarily unavailable. Under the immediate availability condition, participants rated their likelihood of engaging in immediate unprotected sex versus immediate protected sex using a sliding scale. In the delayed availability condition, they chose between immediate unprotected sex and waiting for a condom to engage in protected sex after varying delays (1 h, 3 h, 6 h, 24 h, 1 week, 1 month, and 3 months). Following the standard SDDT delay sequence used by Johnson et al. (Johnson and Bruner, 2012a, Johnson and Bruner, 2012b)and Hahn et al. (Hahn et al., 2019a, Hahn et al., 2019b), we defined 1 day as the boundary between short-term (≤ 24 h) and long-term (> 1 day) delays, corresponding to the transition in the task's time scale from hours to days/weeks/months.

Responses were recorded on a continuous 0–100 scale (0 = “definitely would have sex,” 100 = “definitely would not have sex”). Based on ratings across delay intervals, the area under the curve (AUC) was calculated following the standard scoring procedures of the Sexual Delay Discounting Task (SDDT) (Johnson and Bruner, 2012a, Johnson and Bruner, 2012b). AUC summarizes the decline in willingness to wait for condom-protected sex across progressively increasing delay intervals. Values range from 0 to 1, with smaller AUC values indicating steeper discounting (greater preference for immediate condomless sex) and larger values indicating lower discounting and greater self-control. AUC is the most commonly used summary index in SDDT research because it provides an overall measure of discounting behavior across delay conditions without requiring assumptions regarding a specific discounting function.

#### 2.4.1 Socio-demographic characteristics

Socio-demographic characteristics, including age, relationship status (single, married, or in a relationship), and monthly income (US$0–2000; US$2001–5000; ≥US$5001), were collected using a self-administered questionnaire.

### 2.5 Statistical analysis

Descriptive statistics (frequencies, percentages, means, and standard deviations) were computed for socio-demographic variables, condom use, substance use, and SDD measures. Based on short-term and long-term AUC scores, a k-means cluster analysis (Wu, 2012) was conducted to classify participants into four subgroups: (1) Globally impulsive group (low short-term and long-term AUC scores), (2) Immediate-impulsive group (low short-term, high long-term AUC scores), (3) Delayed-impulsive group (high short-term, low long-term AUC scores), and (4) High self-control group (high AUC in both domains). These clusters represented distinct patterns of impulsivity across temporal domains. To assess the robustness of the cluster solution, we conducted bootstrap resampling (*n* = 1000), which yielded a mean adjusted Rand index (ARI) of 0.89 (95% CI: 0.67–1.00)(Rand, 1971). The four-cluster solution also demonstrated acceptable separation, with a mean silhouette coefficient of 0.56, indicating good cluster stability and reproducibility(Rousseeuw, 1987).

Ordinal logistic regression model (Harrell Jr, 2015)was then fitted to examine the associations of substance use (including the alcohol, illicit drug, and prescription medication use) and SDD group with CAS. Moreover, interaction teams between substance use and SDD grouping (using three dummy variables) were included to test whether SDD grouping moderate the relationship between substance use and CAS. All continuous predictors were mean centered prior to interaction analyses. All models controlled for socio-demographic covariates. Statistical analyses were conducted using R version 4.5.1, and statistical significance was set at *p* < 0.05.

## 3 Results

### 3.1 Sociodemographic and behavioral characteristics

A total of 271 participants were included in the analysis, with a mean age of 24.46 years (SD = 3.60). Most participants were single (89.7%) and reported a monthly income of $0–$2000 (72.3%). With respect to condom use, the majority reported inconsistent use. Substance use patterns varied across alcohol, illicit drug, and prescription medication use. Detailed distributions are presented in Table 1.

**Table 1:** Participants' sociodemographic characteristics and outcome variables.

Variables	n (%)/ M (SD)
Age	24.4 (3.6)
Relationship	
Single	243 (89.7)
In a relationship	28 (10.3)
Monthly income	
$0–$2000	196 (72.3)
$2001–$5000	63 (23.2)
$5001 or more	12 (4.4)
Condom use during the past 90 days	
Never	42 (15.5)
Sometimes	112 (41.3)
About half the time	38 (14)
Most of the time	74 (27.3)
Always	5 (1.8)
Sexual delay discounting group	
Immediate-impulsive group	26 (9.6)
Delayed-impulsive group	60 (22.1)
High self-control group	86 (31.7)
Globally impulsive group	99 (36.5)
Alcohol use during the past 12 months	
Never	66 (24.4)
Less than monthly	79 (29.2)
Monthly	43 (15.9)
Weekly	61 (22.5)
Daily or almost daily	22 (8.1)
Illicit drug use during the past 12 months	
Never	117 (43.1)
Less than monthly	29 (10.7)
Monthly	13 (4.8)
Weekly	42 (15.5)
Daily or almost daily	70 (25.8)
Prescription medication use during the past 12 months	
Never	227 (83.8)
Less than monthly	26 (9.6)
Monthly	5 (1.8)
Weekly	5 (1.8)
Daily or almost daily	8 (3.0)

### 3.2 SDD characteristics and classification

**Fig. 1:**
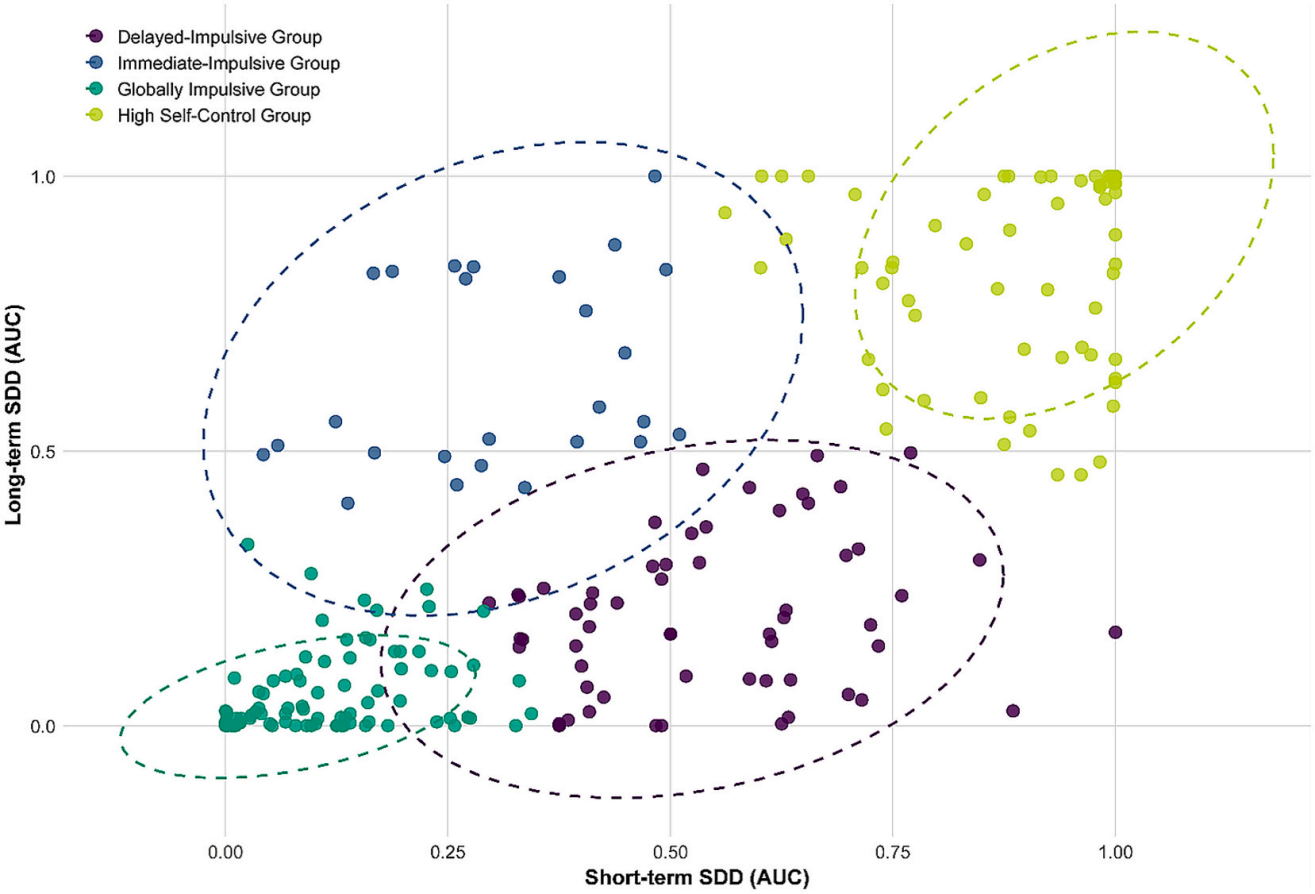
Cluster distribution of SDD based on short-term and long-term AUC values. Note. Each point represents a participant's AUC for short-term (x-axis) and long-term (y-axis) SDD. Higher AUC values indicate greater self-control (less impulsive discounting). Colors represent four SDD groups identified through k-means clustering: High Self-Control, Immediate-Impulsive, Delayed-Impulsive, and Globally Impulsive.

Participants were categorized into four SDD profiles: high self-control, delayed-impulsive, immediate-impulsive, and globally impulsive. Detailed distributions are presented in Table 1 and Fig. 1.

### 3.3 Association of CAS With SDD and substance use

Age and income were not significantly associated with CAS. Being in a relationship was associated with higher odds of CAS compared to single status. None of the substance use variables showed significant main effects on CAS. Compared with the high self-control group, the three impulsive groups showed markedly higher odds of CAS, with the immediate-impulsive and globally impulsive groups showing the strongest effects (Table 2).

**Table 2:** The association between substance use and CAS.

Variables	OR	95% CI	*p*
Age	0.984	0.923, 1.050	0.631
Relationship			
Single	Ref.		
In a relationship	2.624	1.106, 6.324	0.029
Monthly income			
$0–$2000	Ref.		
$2001–$5000	0.850	0.491, 1.469	0.562
$5001 or more	1.009	0.343, 2.933	0.986
Substance use during the past 12 months			
Alcohol use	1.022	0.851, 1.228	0.814
Illicit drug use	1.061	0.920, 1.225	0.415
Prescription medication use	0.820	0.620, 1.082	0.161
Sexual delay discounting group			
High self-control	Ref.		
Delayed-impulsive	2.978	1.598, 5.610	0.001
Immediate-impulsive	8.970	3.814, 21.578	< 0.001
Globally impulsive	9.427	5.179, 17.524	< 0.001

#### 3.3.1 Interactions between SDD groups and substance use

**Fig. 2:**
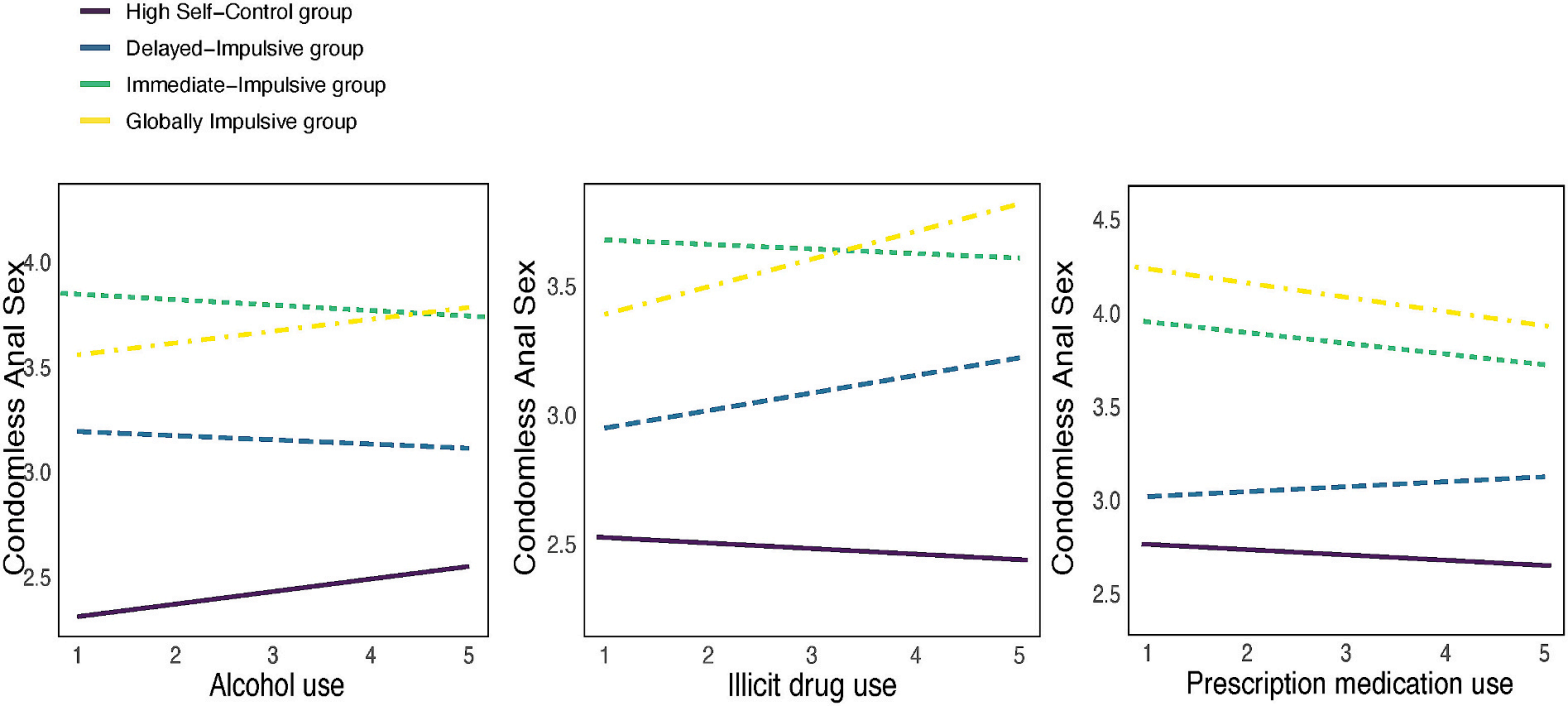
Moderating effects of SDD group across the three substance use models.

The moderation analysis including SDD groups and substance use interactions (see Table 3) showed that alcohol and prescription medication use did not significantly interact with SDD groups. In contrast, in the illicit drug use model using high self-control as reference group in dummy variables, significant positive interactions emerged for the delayed-impulsive (OR = 1.485, *p* = 0.039) and globally impulsive groups (OR = 1.686, *p* = 0.004), suggesting that the association between illicit drug use and sexual risk behavior was amplified among these impulsive subgroups, but not for the immediate-impulsive group (OR = 1.174, *p* = 0.530). The corresponding interaction patterns for variable substance use are illustrated in Fig. 2.

**Table 3:** The moderating effects of SDD grouping on substance use and CAS.

Model	Variables	OR	95% CI	*p*
Model 1				
	Alcohol use	1.138	0.810, 1.609	0.457
	SDD group			
	High self-control	Ref.		
	Delayed-impulsive	3.597	1.899, 6.911	< 0.001
	Immediate-impulsive	10.717	4.576, 25.759	< 0.001
	Globally impulsive	10.736	5.830, 20.237	< 0.001
	Interaction			
	Alcohol use × High self-control	Ref.		
	Alcohol use × Delayed-impulsive	0.862	0.505, 1.467	0.585
	Alcohol use × Immediate-impulsive	0.547	0.284, 1.038	0.067
	Alcohol use × Globally impulsive	0.981	0.629, 1.527	0.934
Model 2				
	Illicit drug use	0.773	0.592, 1.005	0.056
	SDD group			
	High self-control	Ref.		
	Delayed-impulsive	3.544	1.873, 6.799	< 0.001
	Immediate-impulsive	10.014	4.153, 24.756	< 0.001
	Globally impulsive	10.410	5.696, 19.453	< 0.001
	Interaction			
	Illicit drug use × High self-control	Ref.		
	Illicit drug use × Delayed-impulsive	1.485	1.022, 2.164	0.039
	Illicit drug use × Immediate-impulsive	1.174	0.711, 1.941	0.530
	Illicit drug use × Globally impulsive	1.686	1.188, 2.402	0.004
Model 3				
	Prescription medication use	0.743	0.399, 1.411	0.344
	SDD group			
	High self-control	Ref.		
	Delayed-impulsive	3.457	1.829, 6.622	< 0.001
	Immediate-impulsive	9.082	3.815, 22.105	< 0.001
	Globally impulsive	10.381	5.661, 19.461	< 0.001
	Interaction			
	Prescription medication use × High self-control	Ref.		
	Prescription medication use × Delayed-impulsive	1.364	0.616, 2.996	0.435
	Prescription medication use × Immediate-impulsive	1.062	0.471, 2.357	0.881
	Prescription medication use × Globally impulsive	0.816	0.329, 1.955	0.650

Note. All models adjusted for sociodemographic covariates.

## 4 Discussion

This study found that cluster analysis identified four distinct sexual decision-making patterns. Impulsive individuals, particularly those in the immediate-impulsive and globally impulsive groups, were more likely to be associated with unprotected sexual behavior compared with high self-control individuals. Moreover, the delay-impulsive and globally impulsive groups further amplified the association between illicit drug use and unprotected sexual behavior. These findings support the value of adopting a person-centered approach to characterize heterogeneity in sexual delay discounting profiles. Although continuous AUC measures are commonly used in delay discounting research, subgroup-based approaches may capture qualitatively distinct temporal decision-making tendencies that are not fully represented by linear variation alone. By distinguishing between immediate-, delayed-, and globally impulsive profiles, the current findings suggest that impulsive sexual decision-making may vary across temporal contexts rather than existing solely along a single continuum.

This study indicates that individuals in the three impulsive subtypes (global-impulsive, delay-impulsive, and immediate-impulsive) had an elevated risk of engaging in CAS compared with those in the high self-control group, with the immediate-impulsive and globally impulsive group showing particularly high risk. This finding aligns with previous studies using continuous SDD measures(Collado et al., 2017; Dariotis and Johnson, 2015a, Dariotis and Johnson, 2015b; Hahn et al., 2019a, Hahn et al., 2019b) and further confirms that SDD is closely associated with sexual risk behavior among young racial and ethnic minority MSM. A higher level of SDD reflects a stronger preference for immediate rewards; when faced with the choice between immediate sexual gratification and waiting for condom-protected sex, such individuals are more likely to pursue immediate satisfaction [16]. This preference for immediate gratification may be associated with a tendency to prioritize the short-term pleasure of sexual arousal over the long-term health benefits of delayed action. Moreover, insufficient self-control and reduced risk perception may jointly contribute to a greater likelihood of unsafe decision-making under high-arousal conditions (Skakoon-Sparling et al., 2016).

Significant variations were observed in how different SDD groups moderate the link between illicit drug use and CAS. Individuals with delayed impulsive or global impulsive traits showed a stronger association between illicit drug use and sexual risk behavior, consistent with the typical psychological and neural profiles of individuals who use illicit drugs. Individuals who use illicit drugs often exhibit heightened reward sensitivity and weakened delay control, with impulsivity that builds gradually under sustained drug-induced reward activation rather than emerging instantaneously (Volkow & Morales, 2015). The persistent neural reward effect of drugs, characterized by prolonged activation of reward circuits and reduced prefrontal inhibitory control, weakens self-regulation over extended periods [34]. For delayed impulsive individuals, this aligns with their gradual loss of control: as self-regulatory resources decline, ongoing reward stimulation may contribute to risky decision-making. Globally impulsive group, in contrast, display chronically high impulsivity and reward hypersensitivity, which may interact with drug effects and be associated with heightened risk. Meanwhile, immediate impulsive individuals, whose impulsivity is brief and situational (Rezapour et al., 2025), show no significant moderating effect, as their reactions do not overlap with drugs' prolonged neural impact.

The study also found that individuals with higher self-control showed less tendency toward unprotected sexual risk-taking. This suggests that lower delay discounting may protect against risky sexual behavior under drug-induced arousal. These findings have important implications for HIV prevention and behavioral interventions. Targeted interventions for high-SDD individuals, such as delay-of-gratification training(Hudson et al., 2024; Stein et al., 2016), Cognitive Behavioral Therapy(Loya et al., 2023), and Mindfulness-Based Self-Control Training(Bowen et al., 2014), could enhance delay decision-making and inhibitory control, thereby reducing unprotected sex. Incorporating SDD into digital health tools (e.g., mobile apps or online assessments) may further enable personalized and sustainable risk reduction among high-risk MSM populations(Luken et al., 2024).

Contrary to much prior studies, substance use did not demonstrate a significant main effect on CAS in the present study. In the present study, substance use was conceptualized as a relatively stable behavioral tendency rather than an event-level predictor of immediate sexual behavior. From a behavioral economic and dual-process perspective, repeated substance use may reflect enduring alterations in reward sensitivity, impulsive decision-making, and self-regulatory functioning, which may subsequently influence sexual decision-making processes across time(Bechara, 2005; Bickel et al., 2007). Within this framework, sexual delay discounting represents a context-specific manifestation of broader tendencies toward immediate reward preference. Therefore, although substance use and CAS were assessed using different recall windows, the theoretical model assumes that longer-term substance use patterns may remain relevant to more recent sexual risk behavior. Nevertheless, the mismatch in recall periods (past 12-month substance use versus past 90-day CAS) may still have attenuated proximal associations. Additionally, many prior studies have relied on event-level designs that assess substance use immediately preceding sexual activity, thereby capturing more immediate behavioral influences(Hensel et al., 2008; Rendina et al., 2015). Future longitudinal and event-level studies are needed to clarify the temporal sequencing between substance use, delay discounting, and sexual risk behavior.

This study has several limitations. First, it was based on cross-sectional data; although significant associations were identified among SDD, substance use, and CAS, causal inferences cannot be drawn. Future research should employ longitudinal or experimental designs to examine the temporal associations between SDD in substance use and sexual risk behaviors. Second, the data relied primarily on self-reported measures, which may be subject to social desirability and recall biases, potentially underestimating the actual prevalence of risk behaviors. Third, the data were drawn from a larger multi-site study conducted across eight U.S. states and the District of Columbia. As such, the findings may not generalize to MSM from other racial/ethnic backgrounds, lower-risk populations, or individuals residing in other geographic regions. Future research should examine whether similar delay discounting profiles emerge in more diverse and nationally representative samples. Although the cluster solution demonstrated acceptable stability and theoretical interpretability, the separation between clusters was moderate. Therefore, the identified profiles should be interpreted as exploratory temporal impulsivity patterns rather than definitive latent categories. Future studies using larger samples and alternative person-centered approaches, such as latent profile analysis, are needed to further validate these subgroup patterns. CAS was assessed using a single ordinal item on condom use frequency, which may not capture contextual variability in sexual behavior as precisely as event-level or partner-specific measures. Future studies should incorporate more detailed behavioral assessments. In addition, the analyses adjusted for a limited set of demographic covariates. Other psychosocial and structural factors may also have influenced the observed associations. Future studies should incorporate broader psychosocial and structural variables to better account for potential confounding influences. Finally, although illicit drug use was modeled as a composite variable, different substances (e.g., cannabis vs stimulants) may differentially influence sexual decision-making. Future research with larger samples should disentangle substance-specific effects.

## 5 Conclusion

This study suggests that sexual delay discounting is associated with sexual risk behavior among young Black and Latino MSM and may influence how illicit drug use relates to condomless anal sex under specific impulsivity profiles. The observed moderation effects were selective, emerging only for illicit drug use among delayed- and globally impulsive individuals, whereas no significant moderation effects were found for alcohol or prescription drug use. These findings highlight the importance of considering temporal impulsivity in HIV prevention efforts. Future longitudinal research is needed to clarify these associations.

## CRediT authorship contribution statement

**Rui Luo:** Writing – review & editing, Writing – original draft, Visualization, Methodology, Formal analysis, Data curation. **Xiaoming Li:** Writing – review & editing, Methodology. **Xueying Yang:** Writing – review & editing, Methodology.

## Declaration of competing interest

The authors declare that they have no known competing financial interests or personal relationships that could have appeared to influence the work reported in this paper.

## Data Availability

Data will be made available on request.

## References

[bb0005] Amundsen E., Muller A.E., Reierth E., Skogen V., Berg R.C. (2024). Chemsex among men who have sex with men: A systematic scoping review of research methods. Journal of Homosexuality.

[bb0010] Baggaley R.F., White R.G., Boily M.-C. (2010). HIV transmission risk through anal intercourse: Systematic review, meta-analysis and implications for HIV prevention. International Journal of Epidemiology.

[bb0015] Bechara A. (2005). Decision making, impulse control and loss of willpower to resist drugs: A neurocognitive perspective. Nature Neuroscience.

[bb0020] Berry M.S., Johnson M.W. (2018). Does being drunk or high cause HIV sexual risk behavior? A systematic review of drug administration studies. Pharmacology Biochemistry and Behavior.

[bb0025] Bickel W.K., Koffarnus M.N., Moody L., Wilson A.G. (2014). The behavioral-and neuro-economic process of temporal discounting: A candidate behavioral marker of addiction. Neuropharmacology.

[bb0030] Bickel W.K., Miller M.L., Yi R., Kowal B.P., Lindquist D.M., Pitcock J.A. (2007). Behavioral and neuroeconomics of drug addiction: Competing neural systems and temporal discounting processes. Drug and Alcohol Dependence.

[bb0035] Bowen S., Witkiewitz K., Clifasefi S.L., Grow J., Chawla N., Hsu S.H., Lustyk M.K. (2014). Relative efficacy of mindfulness-based relapse prevention, standard relapse prevention, and treatment as usual for substance use disorders: A randomized clinical trial. JAMA Psychiatry.

[bb0040] Collado A., Johnson P.S., Loya J.M., Johnson M.W., Yi R. (2017). Discounting of condom-protected sex as a measure of high risk for sexually transmitted infection among college students. Archives of Sexual Behavior.

[bb0045] Control, C. f. D., & Prevention (2025).

[bb0050] Dariotis J.K., Johnson M.W. (2015). Sexual discounting among high-risk youth ages 18-24: Implications for sexual and substance use risk behaviors. Experimental and Clinical Psychopharmacology.

[bb0055] Dariotis J.K., Johnson M.W. (2015). Sexual discounting among high-risk youth ages 18–24: Implications for sexual and substance use risk behaviors. Experimental and Clinical Psychopharmacology.

[bb0060] Degenhardt L., Webb P., Colledge-Frisby S., Ireland J., Wheeler A., Ottaviano S., Hajarizadeh B. (2023). Epidemiology of injecting drug use, prevalence of injecting-related harm, and exposure to behavioural and environmental risks among people who inject drugs: A systematic review. The Lancet Global Health.

[bb0065] Gebru N.M., Kalkat M., Strickland J.C., Ansell M., Leeman R.F., Berry M.S. (2022). Measuring sexual risk-taking: A systematic review of the sexual delay discounting task. Archives of Sexual Behavior.

[bb0070] Gryczynski J., McNeely J., Wu L.-T., Subramaniam G.A., Svikis D.S., Cathers L.A., Nordeck C.D. (2017). Validation of the TAPS-1: A four-item screening tool to identify unhealthy substance use in primary care. Journal of General Internal Medicine.

[bb0075] Hahn H., Kalnitsky S., Haines N., Thamotharan S., Beauchaine T.P., Ahn W.Y. (2019). Delay discounting of protected sex: Relationship type and sexual orientation influence sexual risk behavior. Archives of Sexual Behavior.

[bb0080] Hahn H., Kalnitsky S., Haines N., Thamotharan S., Beauchaine T.P., Ahn W.-Y. (2019). Delay discounting of protected sex: Relationship type and sexual orientation influence sexual risk behavior. Archives of Sexual Behavior.

[bb0085] Harrell F.E. (2015). Regression modeling strategies: With applications to linear models, logistic and ordinal regression, and survival analysis.

[bb0090] Health N.I. (2026).

[bb0095] Hensel D.J., Fortenberry J.D., Orr D.P. (2008). 2: Event-level substance use and condom use among adolescent women. Journal of Adolescent Health.

[bb0100] Hibbert M.P., Hillis A., Brett C.E., Porcellato L.A., Hope V.D. (2021). A narrative systematic review of sexualised drug use and sexual health outcomes among LGBT people. International Journal of Drug Policy.

[bb0105] Hudson J.E., Grunevski S., Sebelius J., Yi R. (2024). Art-delivered episodic future thinking reduces delay discounting: A phase IIa proof-of-concept trial. Journal of Substance Use and Addiction Treatment.

[bb0110] Jarmolowicz D.P., Hudnall J.L., Hale L., Fowler S.C., Bortolato M., Lemley S.M., Sofis M.J. (2017). Delay discounting as impaired valuation: Delayed rewards in an animal obesity model. Journal of the Experimental Analysis of Behavior.

[bb0115] Johnson M.W., Bruner N.R. (2012). The sexual discounting task: HIV risk behavior and the discounting of delayed sexual rewards in cocaine dependence. Drug and Alcohol Dependence.

[bb0120] Johnson M.W., Bruner N.R. (2012). The sexual discounting task: HIV risk behavior and the discounting of delayed sexual rewards in cocaine dependence. Drug and Alcohol Dependence.

[bb0125] Johnson M.W., Bruner N.R. (2013). Test–retest reliability and gender differences in the sexual discounting task among cocaine-dependent individuals. Experimental and Clinical Psychopharmacology.

[bb0130] Johnson M.W., Strickland J.C., Herrmann E.S., Dolan S.B., Cox D.J., Berry M.S. (2021). Sexual discounting: A systematic review of discounting processes and sexual behavior. Experimental and Clinical Psychopharmacology.

[bb0135] Kelley C.F., Kraft C.S., De Man T.J., Duphare C., Lee H.-W., Yang J., Sullivan P.S. (2017). The rectal mucosa and condomless receptive anal intercourse in HIV-negative MSM: Implications for HIV transmission and prevention. Mucosal Immunology.

[bb0140] Loya J.M., Benitez B., Kiluk B.D. (2023). The effect of cognitive behavioral therapy on impulsivity in addictive disorders: A narrative review. Current Addiction Reports.

[bb0145] Luken A., Rabinowitz J.A., Wells J.L., Sosnowski D.W., Strickland J.C., Thrul J., Maher B.S. (2024). Designing and validating a novel method for assessing delay discounting associated with health behaviors: Ecological momentary assessment study. JMIR formative research.

[bb0150] Lukinova E., Erlich J.C. (2021). Quantifying the contribution of individual variation in timing to delay-discounting. Scientific Reports.

[bb0155] Maisto S.A., Palfai T., Vanable P.A., Heath J., Woolf-King S.E. (2012). The effects of alcohol and sexual arousal on determinants of sexual risk in men who have sex with men. Archives of Sexual Behavior.

[bb0160] Matta A.d., Gonçalves F.L., Bizarro L. (2012). Delay discounting: Concepts and measures. Psychology & Neuroscience.

[bb0165] Maxwell S., Shahmanesh M., Gafos M. (2019). Chemsex behaviours among men who have sex with men: A systematic review of the literature. International Journal of Drug Policy.

[bb0170] Odum A.L. (2011). Delay discounting: I’m ak, you’re ak. Journal of the Experimental Analysis of Behavior.

[bb0175] Rand W.M. (1971). Objective criteria for the evaluation of clustering methods. Journal of the American Statistical Association.

[bb0180] Rendina H.J., Moody R.L., Ventuneac A., Grov C., Parsons J.T. (2015). Aggregate and event-level associations between substance use and sexual behavior among gay and bisexual men: Comparing retrospective and prospective data. Drug and Alcohol Dependence.

[bb0185] Rezapour T., Nafissi N., Rafei P., Vassileva J., Ekhtiari H. (2025). Context-dependent impulsivity in substance use disorder: A neuroscience-informed framework. Current Addiction Reports.

[bb0190] Rousseeuw P.J. (1987). Silhouettes: A graphical aid to the interpretation and validation of cluster analysis. Journal of Computational and Applied Mathematics.

[bb0195] Scott-Sheldon L.A., Carey K.B., Cunningham K., Johnson B.T., Carey M.P., Team M.R. (2016). Alcohol use predicts sexual decision-making: A systematic review and meta-analysis of the experimental literature. AIDS and Behavior.

[bb0200] Skakoon-Sparling S., Cramer K.M., Shuper P.A. (2016). The impact of sexual arousal on sexual risk-taking and decision-making in men and women. Archives of Sexual Behavior.

[bb0205] Stafylis C., Vavala G., Wang Q., McLeman B., Lemley S.M., Young S.D., Revoredo L. (2022). Relative effectiveness of social media, dating apps, and information search sites in promoting HIV self-testing: Observational cohort study. JMIR formative research.

[bb0210] Stein J.S., Wilson A.G., Koffarnus M.N., Daniel T.O., Epstein L.H., Bickel W.K. (2016). Unstuck in time: Episodic future thinking reduces delay discounting and cigarette smoking. Psychopharmacology.

[bb0215] Sweeney M.M., Berry M.S., Johnson P.S., Herrmann E.S., Meredith S.E., Johnson M.W. (2020). Demographic and sexual risk predictors of delay discounting of condom-protected sex. Psychology & Health.

[bb0220] Volkow N.D., Morales M. (2015). The brain on drugs: From reward to addiction. Cell.

[bb0225] Wu J. (2012). Advances in K-means clustering: A data mining thinking.

[bb0230] Zauberman G., Kim B.K., Malkoc S.A., Bettman J.R. (2009). Discounting time and time discounting: Subjective time perception and intertemporal preferences. Journal of Marketing Research.

